# Recording and classifying MET receptor mutations in cancers

**DOI:** 10.7554/eLife.92762

**Published:** 2024-04-23

**Authors:** Célia Guérin, David Tulasne

**Affiliations:** 1 Univ. Lille, CNRS, Inserm, CHU Lille, Institut Pasteur de Lille, UMR9020 – UMR1277 - Canther – Cancer Heterogeneity, Plasticity and Resistance to Therapies Lille France; Goethe University Germany; Goethe University Germany

**Keywords:** receptor tyrosine kinase, mutation, targeted therapies, cancer

## Abstract

Tyrosine kinase inhibitors (TKI) directed against MET have been recently approved to treat advanced non-small cell lung cancer (NSCLC) harbouring activating MET mutations. This success is the consequence of a long characterization of MET mutations in cancers, which we propose to outline in this review. MET, a receptor tyrosine kinase (RTK), displays in a broad panel of cancers many deregulations liable to promote tumour progression. The first MET mutation was discovered in 1997, in hereditary papillary renal cancer (HPRC), providing the first direct link between MET mutations and cancer development. As in other RTKs, these mutations are located in the kinase domain, leading in most cases to ligand-independent MET activation. In 2014, novel MET mutations were identified in several advanced cancers, including lung cancers. These mutations alter splice sites of exon 14, causing in-frame exon 14 skipping and deletion of a regulatory domain. Because these mutations are not located in the kinase domain, they are original and their mode of action has yet to be fully elucidated. Less than five years after the discovery of such mutations, the efficacy of a MET TKI was evidenced in NSCLC patients displaying MET exon 14 skipping. Yet its use led to a resistance mechanism involving acquisition of novel and already characterized MET mutations. Furthermore, novel somatic MET mutations are constantly being discovered. The challenge is no longer to identify them but to characterize them in order to predict their transforming activity and their sensitivity or resistance to MET TKIs, in order to adapt treatment.

## Introduction

MET is a RTK expressed mainly in epithelial cells and activated by its high-affinity ligand, hepatocyte growth factor (HGF). MET activation by HGF promotes a program known as invasive growth, comprising cell division, cell motility, extracellular matrix digestion, invasion, survival, and morphogenic differentiation (Bardelli et al., 1997a; Tamagnone and Comoglio, 1997; Rubin et al., 1993). In an organism, the MET/HGF pair is involved in many processes, including embryonic development (Bladt et al., 1995; Schmidt et al., 1995; Uehara et al., 1995; Maina et al., 1996), development of specific organs/tissues such as the placenta, liver, lung, and muscle (Bladt et al., 1995; Schmidt et al., 1995; Woolf et al., 1995; Yang et al., 1995), and tissue regeneration (Matsumoto and Nakamura, 1992).

**Figure 1.**
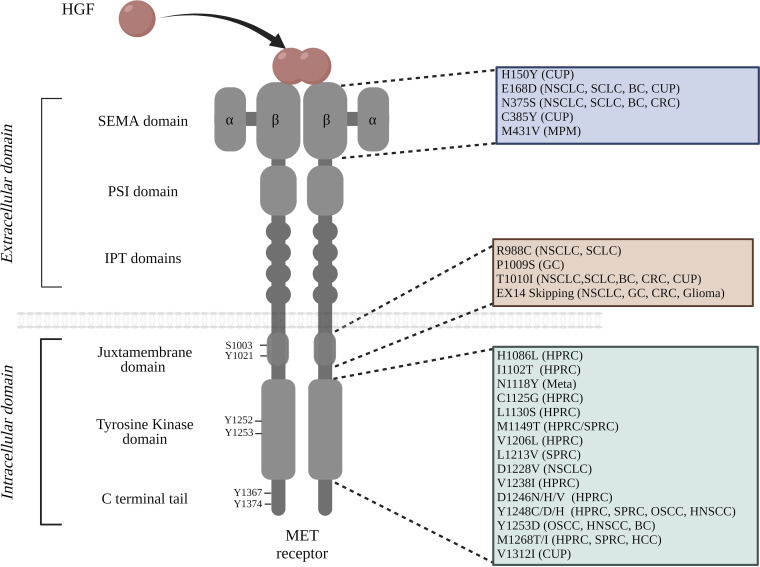
List of MET mutations found within the functional domains of the receptor. The extracellular portion of MET consists of the SEMA domain, a PSI domain, and four immunoglobulin-plexin-transcription (IPT) repeats; the intracellular region contains the juxtamembrane domain, the tyrosine kinase domain, and the carboxyterminal docking site. The cancer types in which particular mutations have been identified are noted in parentheses: breast cancer (BC), cancer of unknown primary origin (CUP), colorectal cancer (CRC), gastric cancer (GC), hepatocellular carcinoma (HCC), hereditary papillary renal carcinoma (HPRC), non-small cell lung cancer (NSCLC), small cell lung cancer (SCLC), sporadic papillary renal carcinoma (SPRC), malignant pleural mesothelioma (MPM), oropharyngeal squamous cell carcinoma (OSCC), and metastasis (Meta). It is worth noting that the amino acid positions are annotated from MET transcript variant 1 NM_001127500.3 (Tovar and Graveel, 2017; Ma et al., 2005; Ma et al., 2003; Liu et al., 2015; Jardim et al., 2014; Stella et al., 2011; Jagadeeswaran et al., 2006; Moon et al., 2000; [Bibr bib91][Bibr bib7]).

MET is synthesized as a precursor which matures through proteolytic cleavage. This leads to the formation of a heterodimeric receptor composed of an extracellular part, containing a SEMA domain, a PSI domain, and four immunoglobulin plexin transcription factor (IPT) domains, and an intracellular part, containing a juxtamembrane domain (JM), a tyrosine kinase domain (TKD), and a C-terminal tail (Figure 1; Stella and Comoglio, 1999; Fernandes et al., 2019; Montagne et al., 2014).

HGF, the high-affinity ligand of MET, is a 90 kDa heterodimeric protein composed of an α subunit with an N domain and four loop domains (domains k1 to k4) and a β chain displaying homology to a serine protease domain (SPH) but lacking catalytic activity (Gherardi et al., 2003). The HGF/MET interaction is ensured by binding the SPH and k1 domains of HGF to the SEMA domain of MET (Gherardi et al., 2006; Uchikawa et al., 2021). However, HGF/MET cryo-EM structure resolutions allow the proposing of different scenarios for MET dimerization. First, MET dimerization could be ensured by HGF dimerization via a top-to-tail interaction of N and k1. This would lead to the association of two MET receptors, each interacting with an HGF. This 2:2 model of interaction suggests a symmetric HGF/MET structure (Gherardi et al., 2006). Second, in a recent study, it has been proposed that one HGF can bind two MET receptors, one through the SPH domain and one through the k1 domain, this leading to MET dimerization. This asymmetric 2:1 model could be further stabilized by heparin and/or binding of a second HGF to the second MET receptor (Uchikawa et al., 2021).

Once the MET receptor interacts with its ligand, it undergoes phosphorylation on two residues of the catalytic domain (Y1252/Y1253 or Y1234/Y1235 in the short MET isoform). It is worth noting that all the mentioned amino acid positions are annotated from the long isoform of MET (MET transcript variant 1 NM_001127500.3). The positions of some amino acids are also mentioned on the basis of the short isoform, as a reminder of the initial publication. Subsequently, other residues outside the catalytic domain are phosphorylated, including residue Y1367/Y1374 (Y1349/Y1356 in the short isoform) in the C-terminal tail, called the multi-substrate docking site (Longati et al., 1994; Ponzetto et al., 1994), as phosphorylated tyrosines in the C-terminal tail are able to recruit multiple effectors such as GRB2-associated binder-1 (GAB1), growth factor receptor–bound protein-2 (GRB2), SRC homology 2 domain-containing (SHC), phosphoinositide 3 kinase (PI3K), non-receptor tyrosine kinase SRC, signal transducer and activator of transcription 3 (STAT3), and phospholipase C-gamma (PLCγ). This multisubstrate docking site plays a key role in the induction by MET of intracellular signalling pathways and biological responses including survival, motility, invasion, and cell cycle progression (Figure 1; Ponzetto et al., 1993; Chiara et al., 2003; Rhoades Smith and Bilen, 2019). In parallel, MET signalling is downregulated by the juxtamembrane region of the cytoplasmic part of the receptor, encoded by exon 14 with its phosphorylated Tyr1021 (Tyr1003 in the short MET isoform) (Cortot et al., 2017).

## Multiple mechanisms of MET activation in cancer

To date, four main genomic events have been described that lead to MET activation and oncogene-driven tumorigenesis (Duplaquet et al., 2018). First, MET gene amplification or copy number gain (CNG), leading to MET overexpression and its ligand-independent activation, has been identified as responsible for carcinogenesis in several tumour types including glioblastoma (Cancer Genome Atlas Research Network, 2023), lung adenocarcinoma (LUAD) (Collisson et al., 2014), gastric cancer (GC) (Asaoka et al., 2010), colorectal cancer (CRC) (Umeki et al., 1999), and medulloblastoma (Tong et al., 2004). The phenomenon was observed for the first time in gastric carcinoma, where MET overexpression caused by gene amplification led to its activation and to the transformation of several cell lines (Ponzetto et al., 1991). Second, HGF overexpression has been observed in breast, gastric, colon, and lung cancers and leads to aberrant MET activation through establishment of an autocrine loop (Ujiie et al., 2012). The third mechanism, found in a limited number of tumours, is chromosomal rearrangement leading to MET fusion with another gene. This leads notably to constitutive MET activation or overexpression and involves fusions including KIF5B-MET in LUAD and BAIAP2L1-MET and C8ORF34-MET in RCC (Stransky et al., 2014). The fourth type of genomic event responsible for MET activation is MET-activating mutations, which constitute the main focus of this review. Since the first MET mutation was discovered in HPRC in 1997 (Schmidt et al., 1997), many MET mutations have been identified. The majority of MET mutations were found in papillary renal cancer and are located in the tyrosine kinase domain of MET. Over the last decade, however, other mutations have been identified outside the kinase domain. In NSCLC, for instance, missense mutations have been found in the SEMA and JM domains, in addition to a large panel of mutations leading to exon 14 skipping (Tovar and Graveel, 2017; Ma et al., 2005; Ma et al., 2003; Krishnaswamy et al., 2009; Schrock et al., 2016; Figure 1 and Table 1). Despite all the MET mutations already identified, novel MET mutations are constantly being discovered (Sebai et al., 2022).

**Table 1.** Recording of MET mutations found in cancers (Tovar and Graveel, 2017; Ma et al., 2005; Ma et al., 2003; Liu et al., 2015; Jardim et al., 2014; Stella et al., 2011; Jagadeeswaran et al., 2006; Moon et al., 2000; Park et al., 1999; Bahcall et al., 2016). In this table, recording the MET mutations characterized by functional studies, subdomain localization of MET mutations were indicated as well as their sensitivity to hepatocyte growth factor (HGF) stimulation and the cancer type in which they were identified. The type of functional assay performed to identify them as activating mutations is indicated.

Domain	Subdomain	AA	Cancer	Functional assay	Sensitivity to HGF	References
Extracellular	SEMA	H150Y	CUP	Anchorage-independent growth assay		Stella et al., 2011
		E168D	NSCLC, SCLC, BC, CUP	BaF3 cells, soft agar colony assay SCLC H446		Ma et al., 2003
		N375S	NSCLC, SCLC, BC, CRC	Cell migration, invasion, and colony-forming assay, tumor growth study after cell xenograft		Ma et al., 2005; Jardim et al., 2014
		C385Y	CUP	Anchorage-independent growth assay		Stella et al., 2011
		M431V	Malignant pleural mesothelioma (MPM)	Cell migration and motility assay		Jagadeeswaran et al., 2006
Juxtamembrane		R988C	NSCLC, SCLC	BaF3 cells, soft agar colony assay SCLC H446		Ma et al., 2003; Montagne et al., 2017
		P1009S	GC	Focus formation NIH3T3		Lee et al., 2000
		T1010I	NSCLC, SCLC, BC, CRC, CUP	Focus formation NIH3T3		Lee et al., 2000
Kinase N-lobe		H1086L	HPRC	Focus formation NIH3T3	Yes	Sebai et al., 2022
	P-Loop	I1102T	HPRC	Focus formation NIH3T3	Yes	Sebai et al., 2022
	P-Loop	N1118Y	Metastasis	Cell migration and invasion assay	Yes	Lorenzato et al., 2002
	P-Loop	C1125G	HPRC	Focus formation NIH3T3	Yes	Sebai et al., 2022
	P-Loop	L1130S	HPRC	Focus formation NIH3T3	Yes	Sebai et al., 2022
		M1149T	HPRC/SPRC	Focus formation NIH3T3	Yes	Jeffers et al., 1997; Michieli et al., 1999
Kinase C-lobe		V1206L	HPRC	Focus formation NIH3T3	Yes	Jeffers et al., 1997; Michieli et al., 1999
		L1213V	SPRC	Focus formation NIH3T3	Yes	Michieli et al., 1999
	Activation Loop (1231–1262)	V1238I	HPRC	Focus formation NIH3T3	Yes	Jeffers et al., 1997; Michieli et al., 1999
	Activation Loop (1231–1262)	D1246N/H/V	HPRC	Focus formation NIH3T3	Yes	Jeffers et al., 1997; Michieli et al., 1999; Bahcall et al., 2016
	Activation Loop (1231–1262)	Y1248C/D/H	HPRC, SPRC, OSCC, HNSCC	Focus formation NIH3T3	Yes	Jeffers et al., 1997; Michieli et al., 1999
	Activation Loop (1231–1262)	Y1253D	OSCC, HNSCC, BC	Focus formation NIH3T3		Jeffers et al., 1997; Bardelli et al., 1998; Liu et al., 2015
	COOH-terminal lobe of the kinase domain	M1268T/I	HPRC, SPRC, HCC	Focus formation NIH3T3		Jeffers et al., 1997; Michieli et al., 1999
		V1312I	CUP	Anchorage-independent growth assay		Stella et al., 2011

## MET kinase domain mutations in papillary renal cancer

Despite the numerous deregulations described previously, no direct causal link between MET and cancer progression was evidenced until 1997, when MET-activating mutations were found in HPRC and associated with cancer progression (Schmidt et al., 1997). Renal cell carcinoma is the most common type of kidney cancer in adults. It is a heterogeneous disease composed of multiple subtypes, such as clear cell renal cell carcinoma (80% of RCCs) and non-clear cell renal cell carcinoma, of which the most common subtype is papillary renal cell carcinoma (15–20% of RCCs). Papillary renal cell carcinoma (PRCC) is divided into two subtypes: type I and II, distinguished histologically, type II being the hereditary form (Rhoades Smith and Bilen, 2019; Mendhiratta et al., 2021; Akhtar et al., 2019). Interestingly, HPRC is the first cancer in which MET mutations, segregating from generation to generation, were identified as causal. The first direct link between MET mutations and cancer development was established in 1997 through genomic polymorphism analysis by Schmidt et al., who found the disease to segregate with a locus on chromosome 7q, carrying the MET proto-oncogene. Chromosome-7 duplication, rather than the loss at this location, suggested the involvement of an oncogene suspected of being MET (Schmidt et al., 1997). In addition, because MET belongs to the same family (the receptor tyrosine kinase family) as RET, a proto-oncogene already known to be mutated in other inherited cancer syndromes (Cortot et al., 2017), the MET proto-oncogene was sequenced rather than the two other genes also found at this locus. Through analysis of nine families affected by papillary renal carcinoma and sporadic papillary renal carcinoma, missense mutations of MET were identified, all located in the kinase domain, among which germline mutations (M1149T, V1206L, V1238I, D1246N, and Y1248C) and somatic mutations (D1246H, Y1228C, and M1268T) (Schmidt et al., 1997; Schmidt et al., 1999).

## Consequences of these MET kinase domain mutations

Following this initial identification, several functional studies were performed. Biochemical and biological assays on murine fibroblasts transfected with MET cDNA demonstrated an increase in MET autophosphorylation when mutations were inserted, demonstrating that these are able to cause constitutive activity of the MET receptor (Tovar and Graveel, 2017; Bardelli et al., 1997b; Jeffers et al., 1997; Bardelli et al., 1998; Graveel et al., 2004; Michieli et al., 1999). Interestingly, different levels of tyrosine kinase activity were observed according to the mutation (Jeffers et al., 1998), M1268T substitutions being identified as the most active, followed by Y1248H, L1213V, and D1246H (Jeffers et al., 1997; Figure 1 and Table 1). Next, analysis of focus formation, cell motility, and tumour growth in athymic mice demonstrated a correlation between MET enzymatic activity and the transforming properties of the different mutants (Bardelli et al., 1997a; Schmidt et al., 1997; Bardelli et al., 1998; Graveel et al., 2004). For instance M1268T, the most active mutant, elicited the most marked focus formation (Bardelli et al., 1998). The involvement of HGF stimulation was also investigated. The two most strongly activating variants, M1268T and Y1248H, displayed constitutive activity, being found to transform fibroblasts even in the absence of HGF (Michieli et al., 1999). In contrast, other evaluated mutations (M1149T, V1206L, L1213V, V1238I), displaying weaker activity, still required HGF stimulation for enhanced MET receptor phosphorylation and cell transformation (Chiara et al., 2003; Michieli et al., 1999). These data suggest that transformation mediated by MET mutants can depend or not on HGF, according to the initial level of kinase activation. Interestingly, mutations inducing higher MET phosphorylation (M1268T and Y1248H) occur in the C-terminal lobe of the MET receptor, specifically in or above the activation loop (Jeffers et al., 1997), while the mutations inducing lower phosphorylation (V1206L and M1149T for instance) are located in the N-lobe or near the catalytic site of the kinase domain (between the N and C lobes). This suggests a relation between mutation location and the activation level.

With knock-in mouse models for several germline MET mutations, it has been demonstrated that these MET mutations can induce spontaneous tumorigenesis. This provides strong evidence that activating MET mutations play a key role in promoting tumorigenesis (Graveel et al., 2004; Graveel et al., 2005).

For most MET mutations found in renal cancers, downstream signalling pathway activation has been little or never investigated. For two kinase mutations in the C-terminal lobe, M1268T and Y1248H, an increase in endocytosis/recycling of the receptor to the membrane has been described, along with a decrease in its degradation (Joffre et al., 2011).

## MET mutations in the juxtamembrane domain

While in renal cancers MET mutations are located exclusively in the kinase domain, in lung cancers they mainly affect the juxtamembrane domain, encoded by exon 14. The first juxtamembrane-domain-located mutations to be discovered were point mutations inducing amino acid substitutions within the domain (Seiwert et al., 2009; Lee et al., 2000). These mutations include R988C, P1009S, and T1010I with, for instance, about 1% of NSCLC patients harbouring R988C (Krishnaswamy et al., 2009). Several studies have demonstrated that these amino acid substitutions within the juxtamembrane domain promote migration and colony formation in soft agar and increase levels of reactive oxygen species, a phenomenon liable to participate in cell transformation (Ma, 2015; Jagadeeswaran et al., 2007). Furthermore, it has been shown that they favour the growth of experimental tumours. Whether these mutations are really activating mutations, however, is controversial, since they do not cause MET kinase activation (Lee et al., 2000; Tyner et al., 2010), so their mode of action is not obvious. In addition, recent studies have shown that these mutations can be germline mutations and thus might correspond to polymorphisms ( Tyner et al., 2010; Boland et al., 2013). Nevertheless, strong functional evidence suggests that these mutations, even present in the germline, could favour lung cancer. In the murine strain SWRJ, the presence of the R968C MET variant, corresponding to the human R988C variant, favours the development of lung tumours upon exposure to a carcinogen (Zaffaroni et al., 2005). In addition, we have recently demonstrated that the R988C mutation favours MET proteolytic cleavages by calpain, a cytoplasmic calcium-activated protease (Montagne et al., 2017). This MET cleavage leads to the generation of an intracellular fragment of about 45 kDa (p45 MET), which in epithelial cells promotes migration and invasion induced by HGF stimulation. This suggests an original mechanism of MET activation, independent of activation of its kinase (Montagne et al., 2017). It is worth noting that, in contrast to the p40 MET fragment generated during apoptosis (see next chapter), the p45 MET fragment is unable to amplify apoptosis. This suggests that differential MET processing by proteases generates specialized fragments according to the cell conditions (Fernandes et al., 2019).

## MET mutations leading to exon 14 skipping

**Figure 2.**
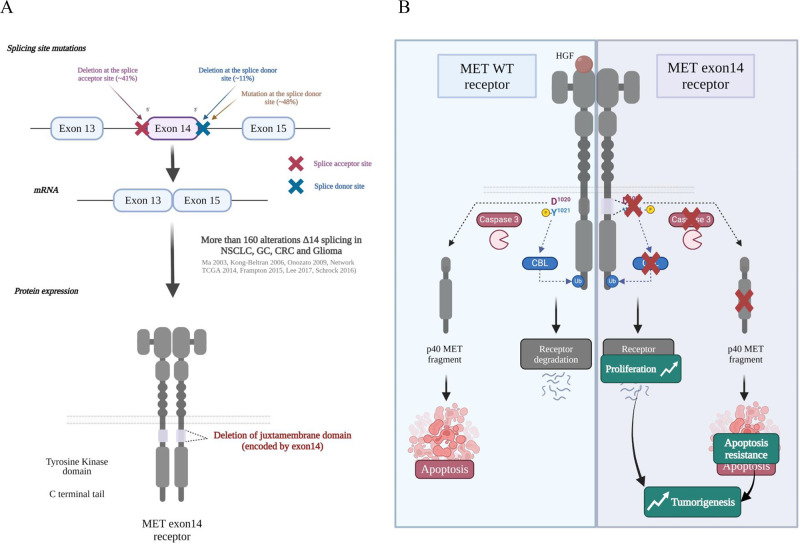
MET mutations leading to exon 14 skipping. (**A**) Several mutations affecting the splice junctions flanking exon 14 (encoding the juxtamembrane domain) have been described in non-small cell lung cancer (NSCLC). More than 160 alterations of the exon 14 splicing sites have been described to induce exon skipping and deletion of the MET juxtamembrane domain (Tovar and Graveel, 2017; Ma et al., 2003; Frampton et al., 2015; Lee et al., 2017). (**B**) Under physiological conditions, MET is also phosphorylated on Tyr1021 in the justamembrane domain, which induces recruitment of Casitas B-lineage lymphoma (CBL), an E3 ubiquitin ligase involved in ubiquitination of the receptor. The ubiquitinated receptor is internalized and degraded, thereby contributing to attenuated signalling. In the absence of its ligand and in response to apoptotic stress, MET is cleaved by caspases to a fragment (p40 MET) able to amplify cell death through permeabilization of the mitochondria. Deletion of the juxtamembrane domain encoded by exon 14 can potentially lead to increased MET stability and tyrosine kinase activity and resistance to cell death. The subsequent increase in proliferation, motility, migration, invasion, and survival promotes tumorigenesis.

Besides mutations causing amino-acid substitutions within the juxtamembrane domain, several mutations affecting the splice junctions flanking exon 14, encoding the juxtamembrane domain, were described in 2006 in two adenocarcinoma tissues and in the NSCLC cell line H596 (Kong-Beltran et al., 2006). After that, a few additional somatic alterations affecting exon 14 were described (Onozato et al., 2009; Vieira et al., 2014), but the rate of these mutations in lung cancers was not determined. In 2014, profiling of lung adenocarcinomas by whole-exome sequencing and mRNA sequencing revealed an approximate 3% rate of somatic mutations leading to exon 14 skipping in lung cancer (Collisson et al., 2014). One year later, the great diversity of MET exon 14 alterations was proved by next-generation sequencing (NGS) across several advanced cancers, including lung, gastric, colorectal, and brain cancers (Frampton et al., 2015). Since then, more than 160 different alterations affecting MET exon 14 have been described, including point mutations, deletions, insertions, and complex mutations (indels) which all affect conserved sequences of donor or acceptor splice sites (Figure 2A; Schrock et al., 2016). At the 5’ end of the exon, most of the alterations are deletions, insertions, and indels affecting the acceptor splice site, the branch point, or the polypyrimidine tract. At the 3’ end, they are mostly point mutations affecting the donor splice site (Figure 2A). Interestingly, all these alterations potentially lead to in-phase exon 14 skipping, as demonstrated by RNA sequencing (Kong-Beltran et al., 2006; Collisson et al., 2014; Davies et al., 2019). In normal mouse liver and kidney, alternative splicing affecting exon 15 (corresponding to exon 14 in human) has been described (exon 15 in transcript ID ENSMUST00000115443). It has been proved that this rare alternative splicing event induces a 141-basepair deletion predicted to result in a 47-amino-acid in-frame deletion in the juxtamembrane region of the cytoplasmic part (Lee and Yamada, 1994). This rare alternative splicing event has not been described, to date, in normal human tissues, but mouse fibroblasts expressing the MET variant with the described deletion display transforming phenotypes and form tumours in immunodeficient mice. This suggests that deletion of exon 15 (corresponding to exon 14 in human) may promote tumorigenesis (Lee et al., 2006; Baek et al., 2004). More recently, in the exon-14-encoded juxtamembrane domain of human MET, several negative regulatory sites have been characterized. The first negative regulatory mechanism found to target the juxtamembrane region was phosphorylation of serine 1003 (Ser985 in the short MET isoform). Ser1003 is phosphorylated by protein kinase C (PKC) and dephosphorylated by protein phosphatase 2 A (PP2A) (Hashigasako et al., 2004). Phosphorylation of MET Ser1003 impairs proper phosphorylation of tyrosine residues, and can thus control cell responsiveness to HGF (Gandino et al., 1994). After liver injury, it has been proved that ligand-dependent activation of the MET kinase, required for tissue regeneration, correlates with decreased Ser1003 phosphorylation (Nakayama et al., 2013). This suggests an involvement of Ser1003 in regulating MET activity in physiological processes. On this basis it would seem that deletion of exon 14 could confer insensitivity to MET-inhibiting signals conveyed by PKC, thus leading to MET activation.

The second identified juxtamembrane-domain residue crucial to MET downregulation is tyrosine 1021 (Tyr1003 in the short MET isoform). Following MET activation, the phosphorylated Tyr1021 residue participates in MET ubiquitination by recruiting the E3 ubiquitin ligase CBL (Casitas B-lineage lymphoma) to the DYpR motif, through interaction with the tyrosine kinase binding domain (TKB) (Peschard et al., 2001; Figure 2B). Interestingly, the Tyr1021 mutation does not impair clathrin-dependent MET internalisation although, if the mutant is internalized, the absence of ubiquitination could prevent its efficient degradation (Li et al., 2007). It has been demonstrated that MET mutated at Tyr1021 promotes more marked cell motility and displays higher stability and a greater transforming capacity than the wild-type version (Weidner et al., 1995; Lai et al., 2012; Abella et al., 2005).

The third regulatory site on exon 14 is a caspase site involved in MET processing during apoptosis. Upon stress induction and in the absence of ligand, the MET receptor is first cleaved at a consensus caspase site in its C-terminal tail. This initial cleavage is followed by a second one, at aspartic acid 1020 (Asp1020) within an ESVD motif of the juxtamembrane domain. This leads to the generation of a 40 kDa cytoplasmic C-terminal fragment (p40 MET) containing the tyrosine kinase domain (Figure 2B; Foveau et al., 2007; Tulasne et al., 2004). During apoptosis, the level of full-length MET receptor decreases drastically after the caspase cleavages. Furthermore, the separation of the extracellular ligand-binding domain from the kinase domain prevents receptor activation by its ligand. Beside the fact that caspase cleavages inactivate the receptor, they also convert it to a pro-apoptotic fragment (p40 MET) capable of stimulating cell death, by promoting calcium flux involved in mitochondrial permeabilization (Lefebvre et al., 2013). Furthermore, in knock-in mice expressing a MET variant mutated at the C-terminal caspase cleavage site, we have highlighted that production of a functional p40 MET is important for FAS-driven hepatocyte apoptosis. Thus, MET acts in vivo as a dependence receptor, through an original signalling mechanism controlling mitochondrial permeabilization (Duplaquet et al., 2020). Caspase cleavages complement the different negative regulatory mechanisms that target this domain and are lost through exon 14 skipping. Interestingly, the ESVD1020 caspase cleavage site overlaps with the DYp1021R recruitment motif for CBL (ESVD1020Yp1021R), and phosphorylation of Y1021 prevents caspase cleavage. This suggests a mechanism protecting phosphorylated MET against caspase cleavage (Deheuninck et al., 2009).

To study the effects of MET exon 14 skipping, investigators have used a clustered regularly interspaced short palindromic repeat (CRISPR/Cas9) system to generate the METex14Del variant in several isogenic cell models (HEK293, H292, and 16HBE cells). A tumour cell line displaying MET exon 14 skipping has also been employed. It appears, firstly, that activation of the METex14Del variant still depends on ligand stimulation. Second, METex14Del induces stronger and more sustained activation of receptor kinase activity and downstream signalling pathways, including the RAS signalling pathway, resulting in significant activation of cell responses such as migration, scattering, and invasion in vitro and metastasis in vivo (Kong-Beltran et al., 2006; Fernandes et al., 2023a; Togashi et al., 2015; Frampton et al., 2015; Lu et al., 2022). Transcriptomic analyses after HGF stimulation have notably revealed expression of matrix metalloproteases known to promote motility and invasion (Cabral-Pacheco et al., 2020) and to be regulated by the RAS signalling pathway (Yang et al., 2014; Tanimura et al., 2003; Genersch et al., 2000). This suggests that sustained activation of RAS-ERK signalling by the METex14Del variant induces expression of MMPs promoting cell motility and invasion.

Only a few functional studies have explored the respective roles of the three known regulatory sites of the juxtamembrane domain in regulating receptor and downstream signalling pathway activation. It seems, however, that the CBL binding site, involved in MET ubiquitination, is a crucial regulatory site (Fernandes et al., 2023b; Miao and Xu, 2019). Furthermore, when METex14Del is present, other known oncogenic drivers such as KRAS (Kirsten rat sarcoma viral oncogene homolog), EGFR (epidermal growth factor receptor), and HER2 (human epidermal growth factor receptor 2) tend to be absent, which suggests that METex14Del can promote oncogenesis in the absence of other oncogenic drivers (Pilotto et al., 2017). The prognostic impact of MET exon 14 skipping has not yet been studied extensively, but previously untreated patients displaying MET dysregulation in NSCLC (due to MET exon 14 skipping, MET amplification, or both) display bad prognosis (Awad, 2016). These results constitute evidence that MET exon 14 skipping alterations are associated, in patients with NSCLC, with poorer outcome. This, combined with their oncogenic driver potential and their prevalence in NSCLC, makes the METex14Del variant an attractive therapeutic target.

## Extracellular MET mutations

Until very recently, the functional consequences of MET mutations affecting the extracellular domain were unknown. Some such mutations were viewed as polymorphisms without any functional consequences. Yet MET N375S, the most common polymorphism known to affect the MET gene and which causes an amino acid substitution in the semaphorin domain, is found also in several cancers, including lung cancers (Tengs et al., 2006). Interestingly, this mutation can confer exquisite binding affinity for HER2, enabling METN375S to interact with HER2 in a ligand-independent fashion. The resulting METN375S/HER2 dimer transduces potent proliferative, pro-invasive, and pro-metastatic cues through the HER2 signaling axis to drive aggressive HNSCC and LUSC cancers (Kong et al., 2020).

## Targeting kinase-domain MET-activating mutations with TKIs

Strategies for inhibiting MET have been developed by pharmaceutical companies and academic laboratories. One approach is to target the HGF-MET interaction with blocking antibodies. Another is to inhibit MET kinase activity with TKIs. In 2013, Choueiri et al tested, in a phase II clinical trial, the compound foretinib, an oral multikinase TKI targeting MET, VEGF, RON, AXL, and TIE-2 receptors in PRCC patients with a MET mutation. Of the patients with a germline MET mutation, five out of ten showed a positive response, while the others showed stable disease. Of the positively responding patients, four showed a 10% reduction of the tumour. Patients without a germline MET mutation showed no such response. Foretinib thus gives rise to a high response rate in patients with a germline MET mutation, with manageable toxicity similar to that of other anti-VEGFR molecules (Choueiri et al., 2013).

In the SAVOIR phase III clinical trial, conducted on 60 patients with chromosomal 7 gain, MET amplification, MET kinase mutation, or HGF alteration, the effect of the MET TKI savolitinib was studied versus sunitinib. Although the study was prematurely terminated, it should be noted that savolitinib showed a response rate numerically superior to that of sunitinib: 27% vs 7%, respectively (Choueiri et al., 2020). Schöffski et al., conducted a phase II clinical trial (CREATE) on type I PRCC patients with MET mutations and MET amplification to test crizotinib. Progression-free survival of more than one year was demonstrated, with durable disease control in 75% of the MET-positive patients (Schöffski et al., 2017).

Finally, combination therapies have also been tested in PRCC. Combining durvalumab, a PD-L1 inhibitor, with the MET inhibitor savolitinib was studied in metastatic RCC patients. For patients with MET impairment, median progression-free survival was 12 months and overall survival was 27.4 months. Combining savolitinib and durvalumab thus seems a possibility for MET-positive tumours (Suárez et al., 2023).

Several other selective and non-selective MET inhibitors are under clinical evaluation, such as INC280 in Phase II for papillary renal cell cancer, crizotinib, and cabozantinib for papillary renal cell carcinoma type 1, and nivolumab, ipilimumab, and tivantinib for papillary renal cell carcinoma type 2 (https://www.clinicaltrials.gov/).

## Targeting METex14Del with TKIs

After initial preclinical studies demonstrating MET TKI efficacy in cell lines displaying MET exon 14 skipping mutations (Togashi et al., 2015; Frampton et al., 2015; Engstrom et al., 2017; Cecchi et al., 2016; Bladt et al., 2013), several clinical trials were performed to evaluate the efficacy of MET TKIs in NSCLC patients displaying MET exon 14 skipping. First clinical evidence of MET exon 14 skipping tumours responding to a MET TKI was obtained in studies using crizotinib, an ATP-binding competitor of anaplastic lymphoma kinase (ALK), c-ros oncogene 1 (ROS1), RON, and MET (Paik et al., 2015; Giroux-Leprieur et al., 2016). This TKI first showed efficacy in several case studies on patients harbouring MET exon 14 skipping tumours (Paik et al., 2015; Drilon et al., 2016; Waqar et al., 2015). Furthermore, data from a clinical trial on a relatively large cohort, in terms of the rate of MET exon 14 mutations (n=69), showed encouraging outcomes in patients with advanced NSCLC and MET exon 14 skipping, with a 9.1 month median duration of response and a 7.3 month median progression free-survival (Drilon et al., 2020). On the basis of these results, in May 2018 crizotinib received breakthrough therapy designation from the United States Food and Drug Administration (US FDA), for the treatment of metastatic NSCLC in patients with MET exon 14 skipping alterations and progression on or after platinum-based chemotherapy (Drilon et al., 2016; Pfizer, 2020). Objective responses to other MET TKIs have likewise been observed, notably in the phase II trial VISION, in which tepotinib was found to induce an objective response in half of the MET exon 14 skipping patients, with an 11 month median duration of response and a 17.1 month median duration of overall survival (Paik et al., 2020). Another MET TKI, capmatinib, gave rise to objective responses in NSCLC patients displaying a MET exon 14 mutation, including previously untreated ones (GEOMETRY trial), with a 12.6 month median response duration. These encouraging results led the Japanese Ministry of Health to approve treatment with TEPMETKO* (tepotinib) for NSCLC patients with MET exon 14 mutations (Merck Global, 2020). At the same time, the US FDA granted approval of capmatinib for treating NSCLC patients harbouring MET exon14 mutations (Novartis, 2020). Combined, the preclinical and clinical evidence proves that patients with MET exon 14 skipping alterations are treatable with MET TKIs, some of which are still under clinical development (Hu et al., 2018; Landi et al., 2019; Yan et al., 2018).

## MET mutations involved in resistance

Over the last few years and as a consequence of the efficacy of MET TKIs in patients harbouring MET exon 14 skipping mutations, several MET TKIs (crizotinib, glesatinib, capmatinib, tepotinib) have been used in the context of clinical trials or as off-label treatments . Yet as expected for targeted therapies, after initial responses, resistances have systematically been observed. Many resistance mechanisms have been described, mostly involving activation of the usual suspects, such as other RTKs or downstream signaling hubs (Jamme et al., 2020; Suzawa et al., 2019). Some resistances, however, are due to on-target mutations, essentially in the MET kinase domain. In the context of this review, we can thus state that MET resists its own targeting.

**Figure 3.**
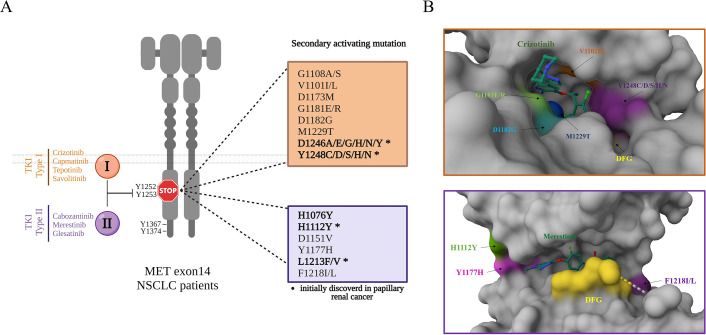
Resistance mutations in the METex14Del variant after treatment with a MET TKI. (**A**) Upon treatment of MET exon 14 skipping NSCLC patients with a type I MET TKI (able to bind the active form of MET kinase) or a type II MET TKI (able to bind the inactive form of MET kinase), secondary resistance mutations were identified. Some of them (*) were already known as activating mutations in papillary renal cancer. Note that the amino acid positions are annotated from MET transcript variant NM_000245.3 (Schmidt et al., 1997; Tovar and Graveel, 2017). (**B**) Secondary activating MET mutations were placed on the 3D structure of the MET kinase domain, along with crizotinib (https://doi.org/10.2210/pdb2WGJ/pdb) or merestinib (https://doi.org/10.2210/pdb4EEV/pdb). MET mutations were inserted into the raw PDB data for MET (Research Collaboratory for Structural Bioinformatics Protein Data Bank DOI: 10.2210/pdb2WGJ/pdb and 10.2210/pdb4EEV/pdb).

The following mutated amino acids have been clinically described after treatment with a MET TKI in patients harbouring MET exon 14 skipping mutations: H1112, G1181, L1213, F1218, D1246, and Y1248, which usually emerge after only a few months of treatment with crizotinib, glesatinib, or capmatinib (Figure 3A and B; Awad et al., 2018; Dong et al., 2016; Guo et al., 2019; Heist et al., 2016; Jin et al., 2019; Li et al., 2017; Ou et al., 2017; Recondo et al., 2020; Rotow et al., 2018; Zhang et al., 2017). The presence of multiple mutations in the kinase domain has also been observed (Awad et al., 2018; Li et al., 2017; Recondo et al., 2020; Rotow et al., 2018; Dong et al., 2016). For example, D1246N/H has been found with Y1248H (Dong et al., 2016) and G1181R can be present with L1213V, D1246H/N, and Y1248H/S (Recondo et al., 2020) in a patient’s circulating DNA.

**Figure 3—video 1.** Resistance mutations of METex14Del treated with MET TKI crizotinib. Video of the three-dimensional crystal structure of the MET kinase domain, along with crizotinib (https://doi.org/10.2210/pdb2WGJ/pdb). Crizotinib (TKI type I) binds the active state (DFG sequence -in) of the kinase. MET resistance mutations were inserted into the raw PDB data for MET (Research Collaboratory for Structural Bioinformatics Protein Data Bank DOI: 10.2210/pdb2WGJ/pdb). The figures and spin structure videos were created with PyMOL software and extracted. The colour code for mutations is the same as in Figure 3.

**Figure 3—video 2.** Resistance mutations of METex14Del treated with MET TKI meristinib. Video of the three-dimensional crystal structure of the MET kinase domain, along with merestinib (https://doi.org/10.2210/pdb4EEV/pdb). Merestinib (TKI type II) binds the inactive stage (DFG-out). MET resistance mutations were inserted into the raw PDB data for MET (Research Collaboratory for Structural Bioinformatics Protein Data Bank DOI: 10.2210/pdb4EEV/pdb). The figures and spin structure videos were created with PyMOL software and extracted. The colour code for mutations is the same as in Figure 3.

In the recent study of Recondo et al., among 20 patients with MET exon 14-skipping mutations who showed resistance to a MET TKI, seven (35%) displayed on-target resistances due to MET mutations, the other resistances being off-target ones (Recondo et al., 2020).

Most of the MET mutations recently found associated with resistance to TKIs were already known as MET-activating mutations, notably in renal cancer (Jeffers et al., 1997; Jeffers and Vande Woude, 1999; Maritano et al., 2000). Detection of these mutations in tumours suggests that they may play a direct role in resistance through ligand-independent MET activation.

Resistance to MET TKIs has also been investigated in cell models displaying MET exon 14 skipping and/or MET exon 14 skipping oncogene dependence. In Ba/F3 cells engineered to depend, for their growth, on ectopic expression of an oncogene rather than on a cytokine, 12 MET resistance mutations were revealed, including five at residues previously spotted in patients (G1181, L1213, F1218, D1246, and T1248) (Fujino et al., 2019). Interestingly, mutations at positions 1246 and 1248 were involved in resistance to type I TKIs, which bind the active form of MET, while mutations at 1213 and 1218 were involved in resistance to type II TKIs, which bind the inactive form. This study thus suggests that the contribution of specific mutations to resistance may depend on the type of TKI (Fujino et al., 2019). These data are also in agreement with the view that a conformational change induced by a particular mutation in the kinase domain may interfere with the binding of particular MET TKIs. For instance, the resistance mutations at positions 1181 and 1248 are consistent with steric hindrance of crizotinib binding (crizotinib being a type I TKI) (Figure 3B; Figure 3—video 1), while the resistance mutations at positions 1213 and 1218 are consistent with steric hindrance merestinib binding, the latter being a type II TKI (Figure 3B; Figure 3—video 2). Interestingly, MET variants with resistance mutations against type I TKIs appear to be sensitive to type II inhibitors, and vice versa (Fujino et al., 2019). This suggests that resistance might be overcome through the complementary activity of different MET TKIs. Similar results have been obtained on NIH3T3 cells: expression of a MET variant displaying both exon 14 skipping and a D1246N or Y1248C mutation was found to drive resistance to type I but not type II inhibitors (Engstrom et al., 2017). In the same publication, the authors report a patient with a MET exon 14 skipping mutation who displayed resistance to crizotinib, associated with the presence of the following additional MET mutations: D1246N, Y1248H/S, and G1181R. On the basis of results obtained on cell models, treatment was switched from the type I inhibitor crizotinib to the type II inhibitor glesatinib. This led to the regression of some metastatic localizations, with disappearance of the Y1248H/S mutation. This disappearance was concomitant, however, with the appearance of an L1213V mutation (Engstrom et al., 2017), identified in a study by Fujino et al., 2019 as leading to resistance to type II TKIs.

On-target resistance has also been described in the context of *MET* gene amplification. A case report describes a patient with an initial EGFR-activating mutation, who developed primary resistance to an EGFR TKI through *MET* amplification (Bahcall et al., 2016). Co-treatment with an EGFR TKI and the MET TKI savolitinib led to a dramatic positive clinical response, but after several months of co-treatment, the patient experienced a relapse associated with detection of an additional MET D1246V mutation, previously described as an activating mutation (Jeffers et al., 1997). In the same study, interestingly, the authors demonstrated with reconstituted MET mutations in cell lines that the D1246V mutant is resistant to type I TKIs (including savolitinib), but not to type II MET inhibitors. This finding led to adapting the treatment: the EGFR TKI was used with cabozantinib, a type II MET TKI. To this treatment, the progressive lung disease responded very well (Bahcall et al., 2016). In a recent study on patients displaying MET gene amplification (following or not treatment with EGFR TKI), treatments with various TKIs led to the appearance of potential resistance mutations, including substitutions at D1246 and Y1248. Interestingly, similar mutations have been found in the context of exon 14 skipping (Yao et al., 2023), suggesting that at least the main on-target resistances are common between the WT MET receptor and the METex14Del variant.

### Conclusion

MET mutations are viewed as drivers if functional data have demonstrated their ability to induce cell transformation and/or experimental tumour growth. To date, several dozen different residues are known to be affected by amino acid substitutions leading to MET activation. Such mutations were first found in the kinase domain and recorded mainly in renal cancers. They lead to kinase domain activation and to induction of downstream intracellular pathways contributing to cell transformation. In various other cancers, other amino acid substitutions have been described within the extracellular domain and the regulatory juxtamembrane domain, but their mechanisms of action remain elusive. The vast majority of MET mutations recorded in lung cancer, in contrast to renal cancer, lead to exon 14 skipping: about 160 different mutations having been recorded (; Ma et al., 2003; Schrock et al., 2016; Frampton et al., 2015; Lee et al., 2017). Mutations leading to amino acid substitutions within the MET kinase have also been found, but to our knowledge none of them have been identified as activating mutations. Thus, although both types of mutation lead to MET receptor activation, their strict differential distribution among cancers (kinase domain for renal cancer and MET exon 14 for NSCLC) suggests that specific organ contexts favour specific transformation mechanisms. For example, it has been shown that METex14Del, in contrast to kinase-domain-mutated MET variants, induces sustained downstream signalling, including RAS-ERK signalling, well known to be activated in lung cancer. In addition, exon 14 skipping leads to loss of the caspase regulatory site and hence to resistance to apoptosis, a property that could also favour tumorigenesis in the lung. An in-depth description of the downstream signalling, transcriptional programs, and biological responses induced by MET kinase mutations and exon 14 skipping would be important in understanding the organ distribution of these MET alterations.

It is worth noting that the METex14Del variant requires HGF stimulation, in keeping with deletion of a regulatory domain (Fernandes et al., 2023a). It thus seems that RTKs with activating mutations may still require ligand stimulation. Likewise, MET variants with activating mutations in the kinase domain could also require ligand stimulation to achieve full receptor activation, as suggested by numerous experimental observations demonstrating that most of the kinase-domain-mutated MET forms recorded in renal cancers can be further stimulated by HGF, with the exception of the form bearing the M1268T mutation (Chiara et al., 2003; Graveel et al., 2004; Michieli et al., 1999; Graveel et al., 2005). Consequently, HGF expression by tumour cells or by the tumour microenvironment could be an attractive biomarker for predicting MET activation and thus its sensitivity to targeted therapies, even in the context of MET mutation.

Recently, treating patients with MET TKIs has led to identifying a novel generation of MET-activating mutations able to cause resistance (Awad et al., 2018; Dong et al., 2016; Guo et al., 2019; Heist et al., 2016; Jin et al., 2019; Li et al., 2017; Ou et al., 2017; Recondo et al., 2020; Rotow et al., 2018; Zhang et al., 2017). These novel mutations are located mainly near the interface between MET and its TKI, easily visible in 3D structures (Figure 3B), and lead to inhibition of TKI binding to its target. Interestingly, some of the resistance mutations discovered are not novel, having already been identified in hereditary and sporadic renal cancer. The fact that recent work has brought historical mutations back to the fore highlights the importance of having an exhaustive record and classification of MET mutations.

With the extensive use of high-throughput sequencing for molecular diagnosis, along with the recent use of targeted therapies against MET, novel MET mutations are thus constantly being discovered. The challenge is no longer to identify MET mutations but to characterize them functionally, in order (1) to identify them as potential targets for therapy, (2) to adapt treatment in case of resistance, (3) to understand why specific classes of mutations are associated with specific cancer types, and (4) to identify novel biomarkers in order to improve targeting of mutated MET.
